# Carotid-cavernous fistula masquerading as thyroid associated orbitopathy: a diagnostic challenge

**DOI:** 10.22336/rjo.2022.33

**Published:** 2022

**Authors:** Mohini Agrawal, Lalitha Kumari, Nitin Vichare, Kripanidhi Shyamsundar, Abhijeet Avasthi, Simple Gupta

**Affiliations:** * Department of Ophthalmology, Command Hospital, Pune, India; ** Department of Ophthalmology, Armed Forces Medical College, Pune, India

**Keywords:** carotid-cavernous fistula, masquerade, proptosis, thyroid-associated orbitopathy, endovascular embolization

## Abstract

**Purpose:** To report a case of indirect carotid-cavernous fistula (CCF) in a patient who presented as a case of thyroid-associated orbitopathy (TAO).

**Case presentation:** A 60-year-old female, known case of hypothyroidism, presented with left-sided headache associated with pain, protrusion and redness of left eye, the examination revealing vision of 20/ 80, proptosis, chemosis and severe ophthalmoplegia. All routine investigations were normal, including thyroid hormone levels. MRI brain & orbits showed increase in bulk of all extraocular muscles with tendon sparing. In view of suspicion of TAO, she was initially misdiagnosed and treated with parenteral and oral steroids, which resulted in further worsening of vision. Optical coherence tomography macula of the left eye revealed acute central serous chorioretinopathy that compelled the stoppage of steroids. While reviewing the patient again, dilated cork-screw tortuous episcleral vessels were found in the left eye. Thus, advised Digital subtraction angiography, confirmed as a case of low-flow left Indirect CCF, managed with endovascular embolization therapy improved her ocular symptoms completely in three days.

**Conclusion:** CCF may mimic TAO due to overlapping features. In-view of different treatment protocols for both, it is critically important to look for atypical features in thyroid eye disease and keep CCF as one of the differential diagnoses for accurate management.

**Abbreviations:** CCF = carotid-cavernous fistula, ICA = internal carotid artery, ECA = external carotid artery, TAO = thyroid-associated ophthalmopathy, BCVA = best corrected visual acuity, MRI = magnetic resonance imaging, IVMP = intravenous methylprednisolone, OCT = Optical coherence tomography, CSCR = central serous chorioretinopathy, DSA = digital subtraction angiography, IOP = intraocular pressure, CT = computed tomography

## Introduction

Carotid-cavernous fistula (CCF) is an abnormal communication between the cavernous sinus and the internal carotid artery (ICA), external carotid artery (ECA) or their branches. It is a sight-threatening condition that may result in complete vision loss. CCFs are divided into two types. Direct high-flow shunts that develop between the ICA and cavernous sinus and is primarily associated with trauma in young males. Whereas, indirect low-flow shunts develop between the cavernous sinus and branches of the ICA, ECA, or both. These are typically spontaneous and seen in older females [**1**]. Though very rare, trauma may also cause formation of indirect CCF and may be associated with hypertension, atherosclerosis, or connective tissue disorders [**2**]. 

CCF can present with varied symptoms like proptosis, blurring of vision, chemosis, headache and ophthalmoplegia. During an early stage, CCF may have atypical manifestations and is prone to being mistakenly identified as other diseases. Many of its symptoms like diplopia, progressive proptosis, conjunctival congestion and chemosis mimic thyroid-associated ophthalmopathy (TAO) in a known case of thyroid disease. While euthyroid does not rule out TAO, specific ocular findings can aid in the correct diagnosis of CCF. Since, both entities differ in their mode of management, the worsening of the signs and symptoms may result in the patient. 

Here, we presented a unique case that was initially misdiagnosed as TAO and subsequently, on management, resulted in the worsening of the symptoms, causing acute central serous chorioretinopathy. Thereafter, revaluation and more digging into relevant clinical findings and investigations facilitated the right diagnosis of CCF. Although they are different entities, the overlapping features of TAO and CCF created a diagnostic challenge. 

## Case description

**Fig. 1:**
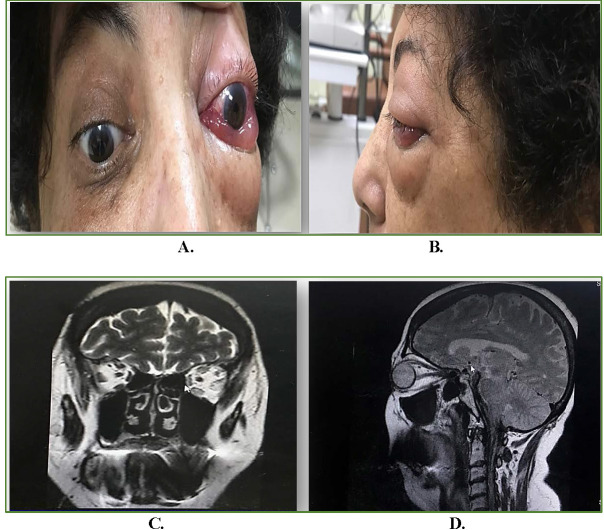
External photograph front view **(A)** and lateral view **(B)** of left eye depicting proptosis, periorbital edema, diffuse conjunctival congestion and chemosis; **C.** Contrast MRI (Coronal view) showing increase in bulk of all the extraocular muscles of left eye; **D.** MRI (Sagittal view) showing increase in bulk of all the extraocular muscles of left eye with tendon sparing

**Fig. 2:**
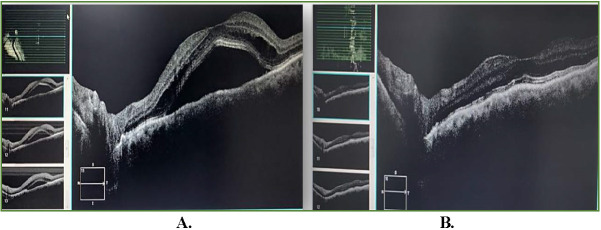
**A.** OCT macula of left eye demonstrating central serous chorioretinopathy post high-dose parenteral steroids; **B.** Resolving central serous chorioretinopathy after stoppage of parenteral steroids

**Fig. 3:**
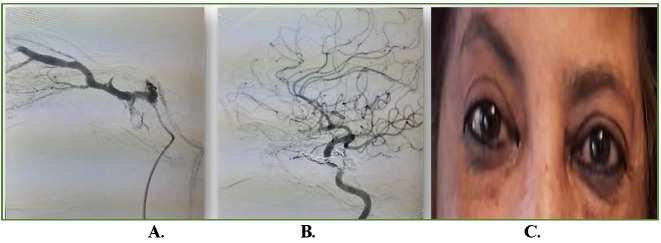
Digital Subtraction Angiography and embolization of left cavernous sinus using platinum coils. **A.** pre-embolization; **B.** post-procedure showing no flow in ophthalmic vein after embolization of left cavernous sinus; **C.** post-operative external photo day 1 after embolization showing improvement of conjunctival congestion, chemosis and proptosis

A 60-year-old female, known case of hypertension and hypothyroidism, since one-year on regular oral medication (Amlodipine and Levothyroxine respectively), presented with left-sided headache associated with pain, protrusion, and redness of left eye of two-weeks duration. Past medical history revealed no history of trauma, fever, weight-loss, or fatigue. On ocular examination, left eye had the best corrected visual acuity (BCVA) of 20/ 80, proptosis (exophthalmos: 20mm right eye and 23mm left eye), periorbital edema, conjunctival congestion, chemosis (**Fig. 1 A, B**), severe ophthalmoplegia, no cells/ flare in anterior chamber and normal posterior segment. Right eye examination was normal. Intraocular pressure was normal in both eyes. On investigations, her levels of thyroid hormones, including anti-thyroid peroxidase antibody levels, were found to be normal, MRI brain and orbits (coronal and sagittal view; **Fig. 1 C, D**) showed an increase in bulk of all extraocular muscles of left eye with tendon sparing. Depending on her clinical picture and imaging, she was misdiagnosed as a case of TAO left eye and started on intravenous methylprednisolone (IVMP-1g/ day for three days), followed by oral steroids on tapering doses. After a few days of steroids, her left eye vision dropped suddenly to counting fingers to two-feet with normal projection of rays. Optical coherence tomography (OCT) macula revealed central serous chorioretinopathy (CSCR) in left eye (**Fig. 2 A**). Hence, the steroids were stopped immediately, which then resulted in dramatic resolution of CSCR on OCT (**Fig. 2 B**). In the view of suspicion of other cause, the patient was re-evaluated and found to have dilated cork-screw tortuous episcleral vessels in the left eye for which a digital subtraction angiography (DSA) was directed to rule out any intracranial vascular abnormality. DSA confirmed a low-flow left indirect CCF (**Fig. 3 A**). Subsequently, she underwent transvenous embolization of the CCF. Check-angiogram showed complete obliteration of the CCF (**Fig. 3 B**). Ocular symptoms dramatically resolved in three days and her vision improved to 20/ 20 post one-week of embolization therapy. She remained ocular symptom free at three months (**Fig. 3 C**).

## Discussion

The clinical assessment of CCF includes ocular pain, proptosis, redness, dilated cork-screw vessels, vision loss, diplopia, restricted ocular motility, increased IOP and ocular bruit [**3**-**5**]. The signs and symptoms of CCF are due to congestion of orbital tissues causing engorgement of orbital veins. Low orbital tissue perfusion pressure and stagnant hypoxia are accountable for tissue edema and restricted ocular movements. 

In fact, TAO presents with similar clinical picture and, in a known case of thyroid disease, the patient is often referred for treatment for the same rather than an unknown diagnosis [**4**]. These patients are often misdiagnosed as having thyroid eye disease or conjunctivitis or blepharitis. In one series, 39% of patients with indirect CCF were initially misdiagnosed as having other ocular diseases, including drop medicamentosa, central venous occlusion and haemorrhagic choroidal detachment [**5**-**7**]. In previous reports like this, TAO was high on the differential initially, even with normal thyroid parameters and CCF that got lurked [**3**,**5**]. It is important for the clinicians to differentiate both entities and note the atypical features in TAO, which should raise the suspicion of other sight-threatening conditions like CCF, as the treatment of both differs.

The treatment protocol used for vision loss in TAO includes steroids, radiation, and surgical orbital decompression, while these options will not improve vision in a case of CCF. Moreover, if not diagnosed correctly, steroids may worsen the vision as seen in our case. Our patient had a dramatic drop in the vision after parenteral steroids and caused acute CSCR, which forced us to stop the treatment then and there. This added to the diagnostic dilemma. Carefully reviewing atypical features that did not fit in TAO guided us to the correct diagnosis. In this case, atypical features included dilated tortuous vessels up to the limbus and worsening of vision on commencement of steroids. Initially, it was important to keep other options open before making it TAO and to consider CCF as one of the differentials. Orbital echography, CT/ MRI scan and cerebral angiography aid in confirming the diagnosis [**5**]. In our case, DSA was found to be an extremely useful investigation to confirm CCF. 

Thus, this case underlines the fact that it is critically imperative to have an open mind for atypical features in TAO and keep CCF as one of the differential diagnoses. The treatment and prognosis of these diseases are two poles apart, therefore, an accurate diagnosis is significant to avert visual morbidity. To the best of our knowledge, our case is unique and first to report the worsening of vision on providing standard treatment for TAO. This standard treatment caused the development of acute CSCR on commencement of steroids in a case of CCF, which was masquerading as TAO. It also stresses upon the importance of timely intervention with endovascular embolization therapy for CCF to prevent visual morbidity.

## Conclusion

Although rare, CCF may mimic TAO by virtue of overlapping features, it should be kept in mind as a differential while treating a case of thyroid eye disease that does not respond to the standard treatment and has unilateral or asymmetric ocular involvement. In-view of different treatment protocols for both, it is critically important to look for atypical features in the thyroid eye disease and keep CCF as one of the differential diagnoses for accurate management.

**Conflict of Interest Statement**

NIL.

The manuscript has been read and approved by all the authors, the requirements for authorship as stated earlier in this document have been met and each author believes that the manuscript represents honest work.

**Informed Consent and Human and Animal Rights statement**

Informed consent has been obtained from the individual included in this study.

**Authorization for the use of human subjects**

Ethical approval: The research related to human use complies with all the relevant national regulations, institutional policies, is in accordance with the tenets of the Helsinki Declaration, and has been approved by the review board of Command Hospital, Pune, India.

**Acknowledgments**

NIL.

**Sources of funding**

NIL.

**Disclosures**

NIL.

**Presentation at a meeting**

NA.
